# Short-term outcomes of anterior cruciate ligament reconstruction with or without lateral tenodesis or anterolateral ligament reconstruction: a retrospective cohort

**DOI:** 10.1007/s00264-023-05931-6

**Published:** 2023-08-26

**Authors:** Ashraf T. Hantouly, Abdulaziz F. Ahmed, Theodorakys Marin Fermin, Luca Macchiarola, Vasileios Sideris, Emmanouil Papakostas, Pieter D’ Hooghe, Khalid Al-Khelaifi, Bruno Olory, Bashir Zikria

**Affiliations:** 1 Department of Orthopedic Surgery, Hamad Medical Corporation, Doha, Qatar; 2 Department of Orthopaedic Surgery, Johns Hopkins School of Medicine, Baltimore, MD USA; 3 Aspetar Orthopaedic and Sports Medicine Hospital, Doha, Qatar; 4 Clinica Ortopedica E Traumatologica II, IRCCS Istituto Ortopedico Rizzoli, Via Pupilli 1, Bologna, BO Italy

**Keywords:** Anterior cruciate ligament, Lateral tenodesis, Anterolateral ligament reconstruction, Extra-articular augmentation

## Abstract

**Purpose:**

This study aimed to compare the short-term outcomes of ACL reconstruction (ACLR) alone, ACLR with lateral tenodesis, and ACL and ALL reconstruction.

**Methods:**

A retrospective cohort of prospectively collected data on all ACL procedures was performed at Aspetar Specialized Orthopaedic and Sports Medicine Hospital between January 2020 and January 2021. Patients were treated with ACLR alone, ACLR with lateral tenodesis, or ACLR with ALL reconstruction. The primary outcome was the subjective International Knee Documentation Committee (sIKDC) score. The secondary outcomes were the ACL Return to Sport after Injury (ACL-RSI) scores, pivot shift grade, subjective knee stability, and subjective pain on activity.

**Results:**

A total of 100 cases were included. The most common technique was ACLR with lateral tenodesis (42%), followed by ACLR alone (38%) and ACL with ALL reconstruction (20%). The mean age was 28.15 years (15–60), and 94% of the patients were males. Meniscal procedures were more frequent in the ACLR alone group (65.8%). There was no association between subjective stability, sIKDC, ACL-RSI, and pivot shift grade and the three ACLR techniques while adjusting for age, sex, and concomitant meniscus procedures at six weeks, 12 weeks, six months, and nine months. However, there was a significant decrease in postoperative flexion in the ACL and ALL reconstruction group by a mean of 22° (95% CI − 40.7 − 3.4; *P* = 0.02) at 6 weeks compared to ACLR alone, which was not evident on later follow-ups.

**Conclusion:**

ACLR with/without lateral augmentation procedures yields similar subjective IKDC, ACL-RSI, pivot shift grade, and subjective knee instability at short-term follow-up. Therefore, lateral extra-articular augmentation procedures are safe to be performed.

## Introduction

Anterior cruciate ligament (ACL) rupture is a severe and debilitating injury for athletes and non-athletes. It is associated with potentially devastating complications such as post-traumatic arthritis and functional limitations. Annually, it is estimated that there are 250,000 ACL injuries in the USA alone, making it the most common ligamentous injury [1, 2]. ACL rupture results in anterolateral and rotational instability, impairs patients’ quality of life, and limits their ability to participate in sports. Therefore, surgical treatment aims to restore knee stability and normal biomechanics to prevent further meniscal and cartilaginous damage, which maximizes the ability to function and return to sports.

Historically, isolated extra-articular tenodesis was the technique of choice for treating ACL ruptures. However, due to the associated limitation in rotational laxity, extra-articular reconstruction was abandoned and replaced by single-bundle and double-bundle intra-articular reconstruction.

Although these conventional reconstruction methods result in good functional outcomes, there is a high re-injury rate, especially in the younger patient, reaching up to 20% [3]. Therefore, some evidence supports that isolated conventional ACL reconstruction is insufficient to restore knee biomechanics, and the need for extra-articular augmentation is mandated. Combining ACL reconstruction with anterolateral ligament reconstruction (ALLR) or lateral tenodesis is proposed as the technique of choice to overcome the limitations of the conventional methods. The additional extra-articular reconstruction works as a secondary stabilizer by offloading the graft and providing a lever arm for rotational support. Nevertheless, there is evidence showing that intra- and extra-articular reconstruction over-constraint may disrupt knee biomechanics, cause graft elongation, and result in early arthritic changes. Therefore, using these combinations is advocated for ACL injuries associated with Segond fracture, high-grade pivot shift, radiographic notch sign, and high-level athletes [4].

Given the mixed results and paucity of evidence regarding the biomechanics and outcomes of extra-articular augmentation of ACL reconstruction, this retrospective cohort study aimed to compare the short-term outcomes of ACL reconstruction (ACLR) alone, ACLR with lateral tenodesis, and ACL and ALL reconstruction.

## Methods and materials

This study was reported using the STrengthening the Reporting of OBservational studies in Epidemiology (STROBE) checklist for cohort studies [5].

### Study design

This study was approved by the Institutional Review Board, and it was conducted at Aspetar Specialized Orthopaedic and Sports Medicine Hospital, Doha, Qatar. It was designed as a retrospective cohort of prospectively collected data on the short-term outcomes of ACL reconstruction (ACLR) alone, ACLR with lateral tenodesis, and ACL and ALL reconstruction. The primary outcome was the subjective International Knee Documentation Committee (sIKDC) score. The secondary outcomes were the ACL Return to Sport after Injury (ACL-RSI) scores, pivot shift grade, subjective knee stability, and subjective pain on activity.

The pivot shift was graded as 0 if absent, 1 if slight subluxation, 2 if a definitive clunk is present, and 3 if subluxation occurs with momentary locking. Subjective knee stability and pain on activity were patient-reported outcome measures on a visual analog-like scale from 0 to 10. The subjective pain scale was 0 if no pain exists on activity and up to 10 if maximal pain is caused by activity. Subjective stability was scored as 0 if total instability is felt by the patient and up to 10 if the patient reported maximum stability.

### Eligibility criteria

The inclusion criteria were adults (age >  = 18 years) who underwent ACLR for an ACL deficient knee, played a competitive sport, and had a pivot shift grade >  = 2. In addition, patients with and without concomitant meniscal procedures were included. Meniscal procedures were debridement, partial meniscectomy, subtotal meniscectomy, or meniscal repair. The exclusion criteria were cases with concomitant realignment osteotomy, knee varus or valgus malalignment > 3°, previous ACLR on either knee, symptomatic knee articular cartilage defect requiring operative treatment other than debridement, and two or more ipsilateral knee ligament injuries.

### Surgical techniques

The ACLRs were performed by five sports medicine fellowship-trained orthopaedic surgeons. At the surgeon’s discretion, the ACL grafts were either hamstrings or bone-patellar tendon-bone (BPTB) autografts. All hamstring autografts were ensured to be of a diameter of 8 mm as a minimum.

Patients were allocated to receive extra-articular augmentation if they had at least one of the following criteria: (1) < 25 years old, (2) generalized hyperlaxity, (3) participation in competitive contact sport, and (4) genu recurvatum > 10°. However, it is important to note that the selection of the specific type of extra-articular augmentation employed (ALL vs. LET) was ultimately determined by the individual surgeon’s preference.

#### Single-bundle ACL

**Fig. 1:**
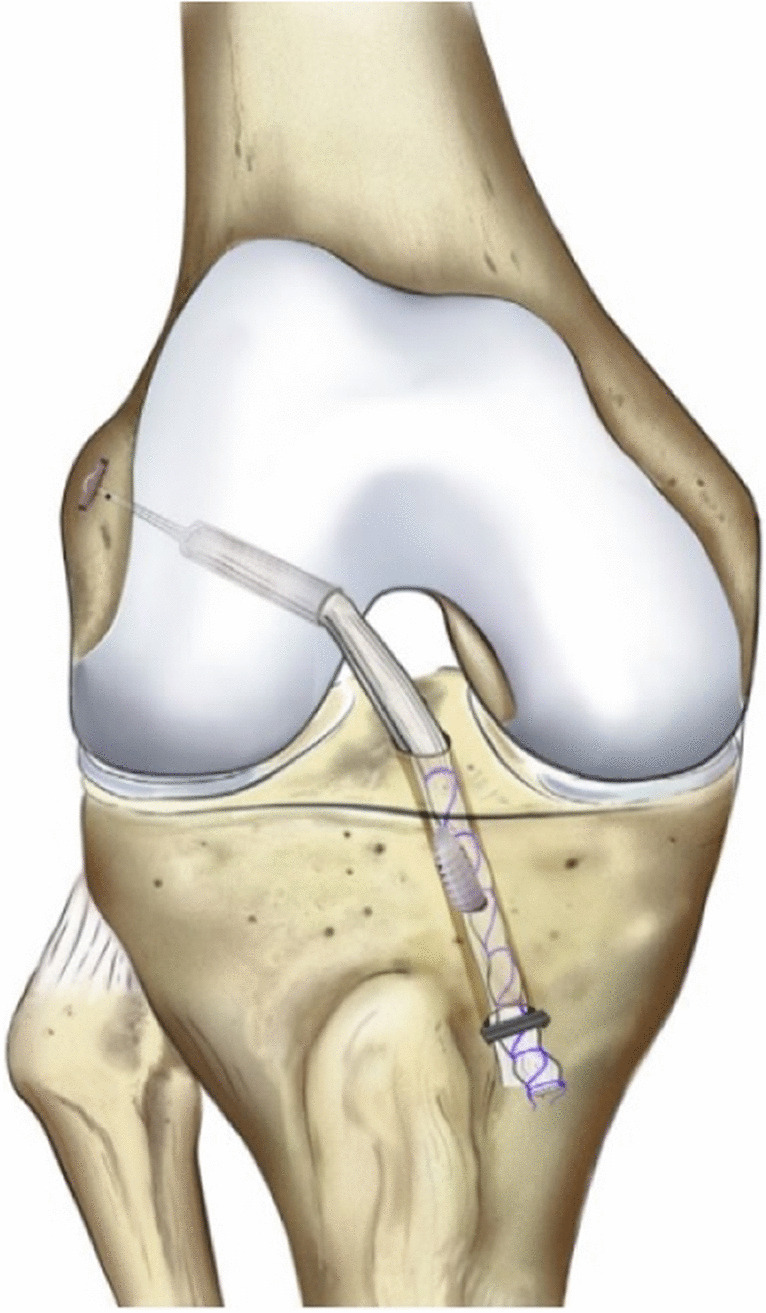
Single-bundle ACL

Hamstring tendons were harvested and detached from the tibial insertion and quadrupled. The starting point of the tibial tunnel was on the medial tibial metaphysis, pointed to the center of the native ACL tibial insertion. A half-tunnel of at least 2.5 cm was drilled in the anatomical center of the femoral ACL footprint, with the knee in flexion [6]. The graft was fixed with a tight rope attachable button system (Arthrex company, Naples USA) against the femoral cortex and in the tibial tunnel using a bioabsorbable interference screw with the knee at 30° of flexion [7] (Fig. 1).

#### ACL + LET

**Fig. 2:**
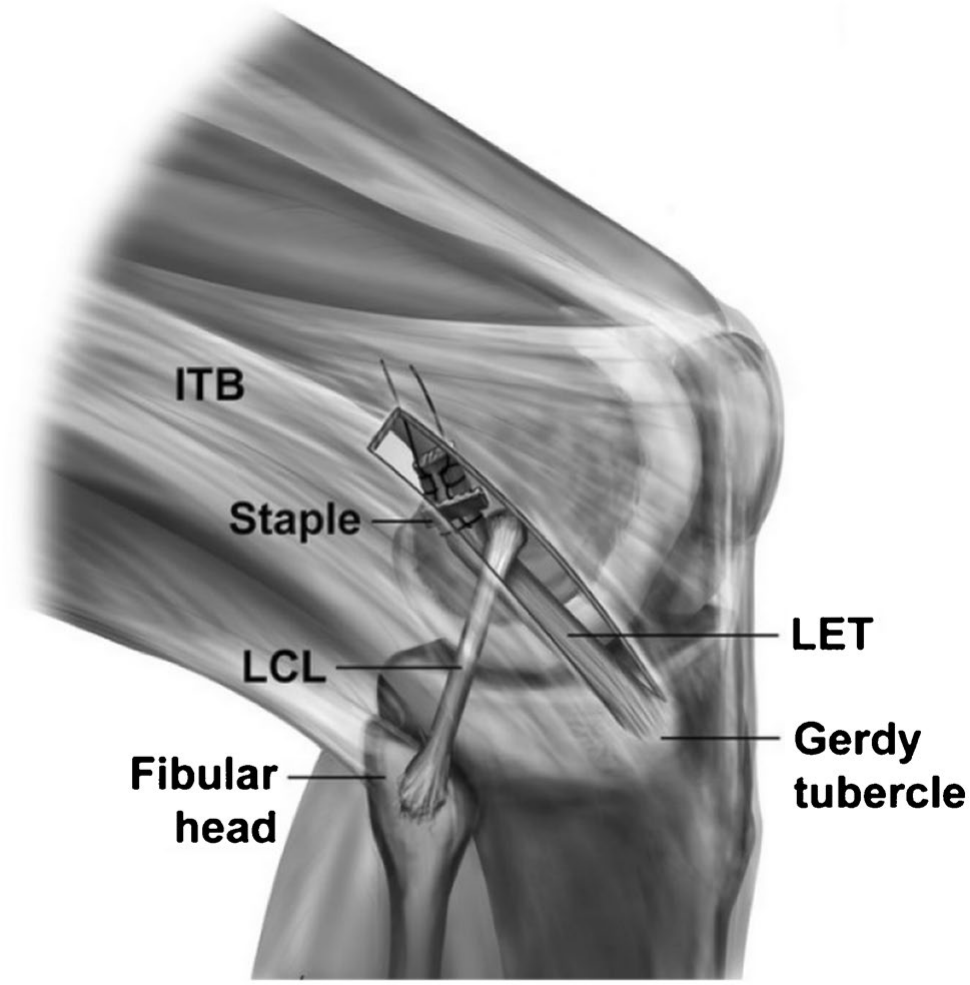
ACL + LET

The ACL was reconstructed with the same technique as the SB-ACL group in this group of patients. At the end of the procedure, a modified Lemaire LET was performed. The skin was incised longitudinally 1 cm posterior to the lateral femoral epicondyle, starting 2 cm proximal to Gerdy’s tubercle; the ITB was harvested in order to obtain a 10–15-cm long by 1-cm wide graft, 1 cm from the posterior edge of the ITB. Then, the graft was passed beneath the lateral collateral ligament (LCL) from distal to proximal using a right-angled clamp; the attachment site was identified around 3 cm higher to gastrocnemius insertion in the posterior third of the femur. Then finally, the graft was secured using a staple or an anchor while at 30° of flexion, based on surgeon preference, and then folded back distally and sutured onto itself [8] (Fig. 2).

#### ACL + ALL

Three bony landmarks were marked: the fibular head, the Gerdy tubercle, and the lateral epicondyle. Two stab incisions 2 cm apart were made between the Gerdy tubercle and the fibular head. One stab incision was made slightly posterior and proximal to the lateral epicondyle on the femur. A 4.5-mm drill bit was used to create a two bony tunnel on the tibia, which were connected subcortically. A suture (no. 1 PDS loop) was then passed retrogradely. The suture was clamped with a haemostat slightly posterior and proximal to the lateral epicondyle at the area previously incised. The knee was then taken through the range of motion to ensure a non-isometry of the ALL graft. It was aimed for tight in extension and slack in flexion. The ST and GR were harvested, maintaining tibial attachment. The ST was measured from its insertion, marked using a pen at 4 cm and 10 cm, then tripled beginning at the proximal and distal marks. The GR was then incised and subsequently sutured at the tripled ST graft. This thus created a tubular ACL graft with 3 parts ST and 1 part GR. (In function of the length of the ST, the final graft could also be quadrupled.)

**Fig. 3:**
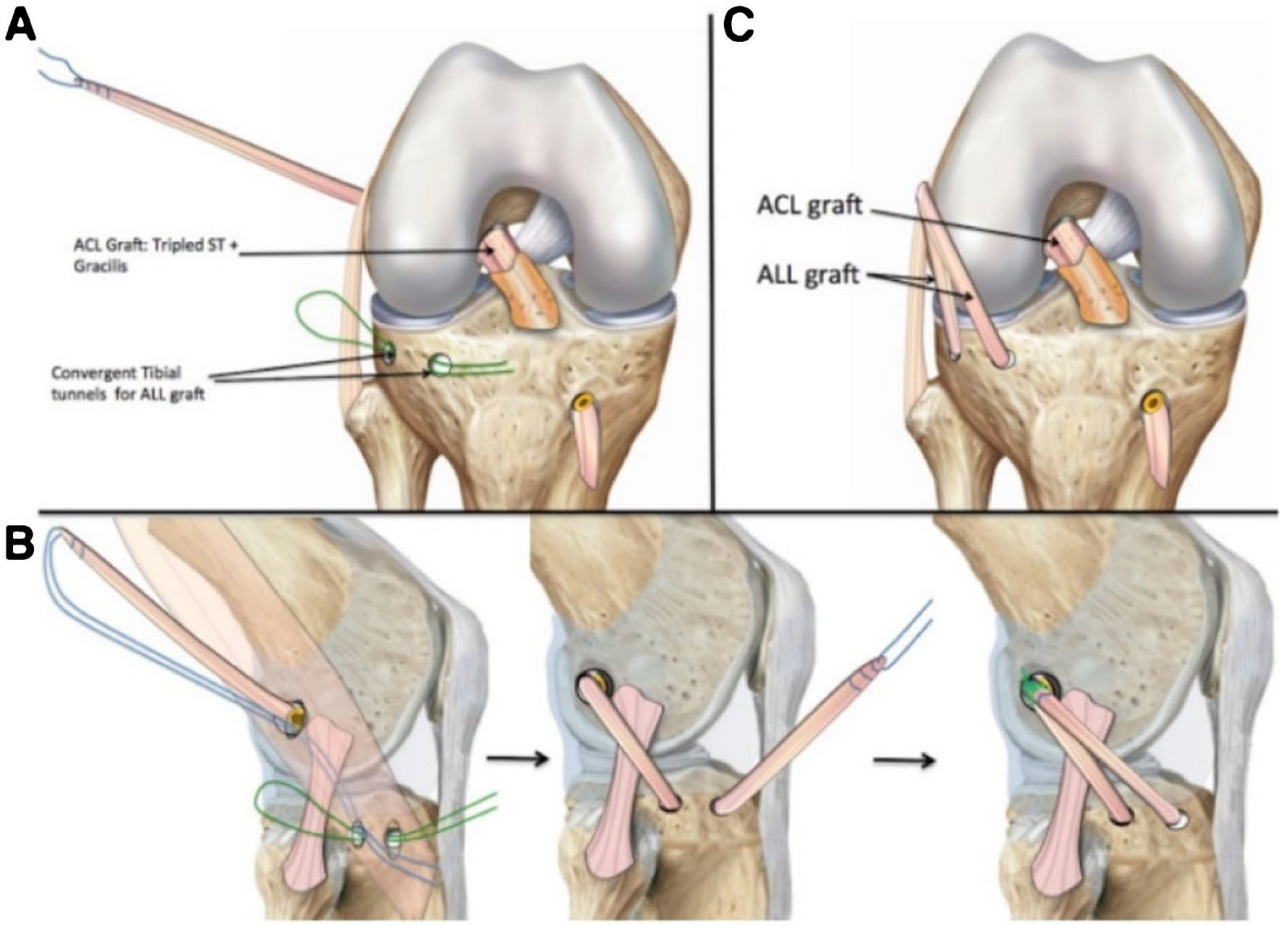
ACL + ALL

The femoral tunnel was performed with an outside-in approach. The intra-articular end of the tunnel was placed at the femoral origin of the ACL, while the cortical end of the tunnel was placed at the appropriate point marked for optimal ALL isometry. The tibial tunnel was drilled in a standard fashion from the external cortex to the ACL insertion. The graft was passed in the tibial and femoral tunnel and out from the lateral femoral cortex. Interference screws, measuring the same size as the ACL graft, were used to secure it. In order to reconstruct the ALL, the suture connected to the remaining length of the GR was shuttled through the tibial subcortical tunnel from posterior to anterior, deep to the iliotibial band, and secured with a 4.75-mm interference screw in full extension. Finally, the graft is brought back proximally out the proximal incision and sutured in full extension [9] (Fig. 3).

### Rehabilitation protocol

A routine ACL rehabilitation program was utilized to all patients regardless of the operative technique, entailing full weight-bearing after the procedure, without a brace, and progressive range-of-motion exercises. Early rehabilitation is focused on obtaining full extension and quadriceps activation. A gradual return to sports activities is allowed starting at four months for non-pivoting sports, at six months for pivoting non-contact sports, and at eight to nine months for pivoting collision sports.

### Data source and collection

The data source was our institution’s electronic medical records. The operative database was searched for all ACL procedures performed from January 2020 to January 2021. The preoperative assessments, operative notes, and postoperative assessments were reviewed.

The baseline variables that were collected included age, sex, sports type, level of competition, ACLR technique (ACLR alone, ACLR with lateral tenodesis, ACL with ALL reconstruction), surgeon, and concomitant meniscus procedures. Outcome variables were patient-reported outcome measures and physician-reported outcome measures. Patient-reported outcome measures were subjective pain on activity, subjective stability, the ACL-RSI, and the sIKDC score. Physician-reported outcome measures were the affected knee flexion, affected knee pivot shift test. The outcomes were assessed at six weeks, three months, six months, and nine months.

### Statistical analysis

The statistical analysis was performed with Stata/IC (StataCorp 2019, Stata Statistical Software: Release 16, College Station, TX: StataCorp LLC). Continuous variables were reported with means and SD, and dichotomous variables were reported as proportions. The analysis of variance test was used to compare age between the three ACLR techniques. Proportions such as sex and the proportion of meniscal procedures were compared with a Fisher exact test. A linear regression model was designed to evaluate the effect of either of the three ACLR techniques on outcomes while adjusting for age, sex, surgeon, and concomitant meniscal procedures. The effects of the three ACLR techniques were estimated as a mean difference at a 95% confidence interval (CI). In addition, a difference with a *P* value < 0.05 was considered statistically significant.

## Results

### Participants

A total of 100 ACLR cases were eligible for inclusion in this study period between January 2020 and January 2021. The most common technique was ACLR with lateral tenodesis in 42%, followed by ACLR alone in 38% and ACL with ALL reconstruction in 20%. The mean age was 28.15 years (15–60), with most patients being males (94%). The most common sport was soccer (62.2%), followed by handball (6.7%) and volleyball (4.4%). Meniscal procedures, mainly in the form of partial meniscectomy or meniscal repair, were performed in 45% of cases. Meniscal procedures were more frequent in the ACLR alone group (60.5%) compared to the two other ACLR techniques. Out of 100 patients, follow-up was completed in 65% of patients at six weeks, 44% of patients at 12 weeks, 45% at six months, and 24% of patients at nine months postoperatively. Of note, none of the ACL with ALL reconstruction patients were available for follow-up at the six and nine month follow-up intervals, as this technique was adopted recently at our institution. Table 1 summarizes the patients’ characteristics.

**Table 1:** Patients’ characteristics

	ACLR alone (*N* = 38)	ACLR + LT (*N* = 42)	ACLR + ALLR (*N* = 20)	*P* value
Age mean age + / − SD	30.4 + / − 11.6	24.6 + / − 9	31.2 + / − 10.3	0.29*
Sex *n* (%) Male Female	35 (92.1%) 3 (7.9%)	40 (95.2%) 2 (4.8%)	19 (95%) 1 (5%)	0.87^†^
Sports *n* (%)	10 soccer (83.3%) 1 martial art (8.3%) 1 volleyball (8.3%)	13 soccer (59.1%) 2 handball (9.1%) 1 basketball (4.6%) 1 volleyball (4.6%) 1 equestrian (4.6%) 1 tennis (4.6%) 1 table tennis (4.6%) 1 rugby (4.6%) 1 equestrian (4.6%)	6 soccer (54.5%) 1 handball (9.1%) 1 archery (9.1%) 1 cycling (9.1%) 1 marine sports (9.1%) 1 modern pentathlon (9.1%)	–
Level of participation *n* (%) Competitive Intermediate Recreational Others	6 (15.8%) 10 (26.3%) 15 (39.5%) 7 (18.4%)	7 (16.7%) 18 (42.9%) 9 (21.4%) 8 (19%)	2 (10%) 10 (50%) 5 (25%) 3 (15%)	–
Graft type	HT 28 BTP 9 QT 1	HT 29 BPTB 12 QT 1	HT 20	0.052^†^
Type of meniscus procedure				
Partial medial meniscectomy	4	5	0	–
Partial lateral meniscectomy	3	1	0	–
Partial medial and lateral meniscectomy	0	1	0	–
Medial meniscus repair	6	1	0	–
Lateral meniscus repair	6	7	4	–
Medial and lateral menisci repair	2	2	0	–
Subtotal meniscectomy	1 (medial meniscus) 1 (lateral meniscus)	1 (medial & lateral menisci)	0	–
Total *n* (%)	23 (60.5%)	18 (42.9%)	4 (20%)	0.001^†^

*ACLR* anterior cruciate ligament reconstruction, *LT* lateral tenodesis, *ALLR* anterolateral ligament reconstruction, *SD* standard deviation, *HT* hamstring tendon, *BPTB* bone patellar tendon bone, *QT* quadriceps tendon

^*^Analysis of variance statistical comparison

^†^Fisher’s exact statistical comparison

### Patient-reported outcome measures

Table 2 summarizes the postoperative outcome measures. Regarding the sIKDC, linear regression analyses demonstrated no association between sIKDC and the three ACLR techniques while adjusting for age, sex, concomitant meniscus procedures, and surgeon at six weeks, 12 weeks, six months, and nine months. Likewise, adjusted linear regression analyses demonstrated no association between ACL-RSI and the three ACLR techniques at six weeks, 12 weeks, six months, and nine months.

**Table 2:** Summary of outcome measures following anterior cruciate ligament reconstruction (ACLR) alone, ACLR with lateral tenodesis (LT), and ACLR with anterolateral ligament (ALLR) reconstruction

	Subjective IKDC	ACL-RSI	Knee flexion	Pivot shift grade	Subjective pain	Subjective stability
6 weeks						
ACLR alone (*N* = 19)	44.6 + / − 12.2	853.7 + / − 256.3	127° + / − 12	N/A	2 + / − 2.8	7.5 + / − 1.4
ACLR + LT (*N* = 29)	42 + / − 8.4	761.4 + / − 276.1	125.8° + / − 17.2	N/A	2 + / − 1.9	7.9 + / − 2
ACLR + ALLR (*N* = 17)	45.8 + / − 9.7	850 + / − 223	112.2° + / − 24	N/A	2 + / − 2	8.7 + / − 2
12 weeks						
ACLR alone (*N* = 13)	57.4 + / − 12.6	967.7 + / − 147.9	130° + / − 13.5	0.6 + / − 0.9	2 + / − 2.2	8.4 + / − 1.9
ACLR + LT (*N* = 20)	55.9 + / − 12.2	855 + / − 232.4	132.3° + / − 10.3	0.5 + / − 0.68	1.3 + / − 1.5	8.3 + / − 2
ACLR + ALLR (*N* = 11)	58.5 + / − 12	999.1 + / − 188.8	129.3° + / − 9	0.3 + / − 0.48	2.1 + / − 2	9.3 + / − 1.4
6 months						
ACLR alone (*N* = 20)	67.95 + / − 13.9	866 + / − 263.5	130° + / − 24.5	0.7 + / − 1.1	0.6 + / − 1.1	8.3 + / − 1.7
ACLR + LT (*N* = 24)	63.7 + / − 10.3	907 + / − 195.6	134.2° + / − 11.3	0.4 + / − 0.7	0.8 + / − 1.4	9.1 + / − 1.5
ACLR + ALLR (*N* = 0)	N/A	N/A	N/A	N/A	N/A	N/A
9 months						
ACLR alone (*N* = 10)	68 + / − 13.2	809 + / − 266.4	150.8° + / − 5.9	0	1 + / − 1.4	8.6 + / − 1.1
ACLR + LT (*N* = 14)	69.6 + / − 12	994.3 + / − 189.1	138.8° + / − 3.8	0.1 + / − 0.4	1.3 + / − 1.7	9.5 + / − 0.8
ACLR + ALLR (*N* = 0)	N/A	N/A	N/A	NA	N/A	N/A

*SD* standard deviation, *IKDC* international knee documentation committee, *ACL-RSI* anterior cruciate ligament return to sports after injury

In terms of subjective pain, no association was found between the three ACLR techniques while adjusting for age, sex, and surgeon at six weeks. However, performing a concomitant meniscus procedure decreased the mean pain score by 1.1 points (95 CI − 2.1 − 0.1; *P* = 0.03) at six weeks. Longer follow-up points at 12 weeks, six months, and nine months failed to demonstrate any association between subjective pain and the three ACLR techniques. Similarly, the subjective stability was not affected by any ACLR techniques while adjusting for age, sex, concomitant meniscus procedures, and surgeon at six weeks, 12 weeks, six months, and nine months.

### Physician-reported outcome measures

In terms of postoperative knee flexion, linear regression analyses demonstrated a statistically significant decrease of postoperative flexion in the ACL and ALL reconstruction group by a mean of 22° (95% CI − 40.7 − 3.4; *P* = 0.02) at six weeks compared to ACLR alone. There was no statistically significant difference for knee flexion between ACLR alone and ACL with lateral tenodesis or between ACLR with lateral tenodesis and ACL with ALL reconstruction at six weeks. In contrast, no differences in mean knee flexion between any of the techniques were found at 12 weeks, six months, and nine months. Moreover, no statistically significant difference in knee flexion was found when accounting for age, sex, concomitant meniscus procedures, and surgeon.

No differences in the pivot shift grade were found between the three ACLR techniques on adjusted linear regression models for age, sex, concomitant meniscus procedures, and surgeon at six weeks, 12 weeks, six months, and nine months.

## Discussion

The most relevant finding of the present retrospective cohort study is that no significant differences were found in terms of subjective IKDC, ACL-RSI, pivot shift grade, and subjective knee instability among the three ACLR techniques at short-term follow-up. Additionally, two significant differences were found at six week follow-up: (1) a mean decrease of 1.1 in the subjective pain on activity score in patients undergoing concomitant meniscus procedure and (2) a mean decrease of 22° of flexion in the ACL with ALL group when contrasted to the isolated ACLR group.

Several studies have evaluated the effects of lateral augmentation techniques, ALL or LET reconstruction. Recently, a biomechanical study by Delaloye et al. showed that both lateral augmentation techniques were able to restore anterior tibial translation and internal rotation similar to its native state in ACL with anterolateral structure-deficient knees with no significant differences. Additionally, no over-constraint was observed with neither technique [10].

Meta-analyses have also supported these findings by comparing clinical outcomes of isolated ACLR and both techniques. In a meta-analysis conducted by Na et al. including twenty studies with a mean of 42-month (6 months to 19.8 years) follow-up, the comparison between isolated ACLR and ACLR with either lateral augmentation technique, ALL or LET, revealed improved pivot shift tests and IKDC, Lysholm, and Tegner scores regardless of the augmentation technique or time from injury to surgery when compared to the isolated ACLR [11].

Similarly, a meta-analysis by Beckers et al. comprising eleven studies comparing isolated ACLR versus ACLR with lateral augmentation techniques with a minimum two year follow-up found a higher rotational laxity in the isolated ACLR when compared to ACLR with lateral augmentation through pivot shift testing. Likewise, a reduction in anterior tibial translation with the Lachman test and KT-1000 arthrometer was noted when lateral augmentation was added. Nevertheless, there were no differences in IKDC, Lysholm, and Tegner scores and return to sports [12].

These studies support lateral augmentation techniques in ACLR to improve translational and rotational ACL stability and clinical outcomes and reduce the risk of graft rupture [11, 12]. In our cohort, no differences were found in terms of rotational stability between the three groups. Many plausible explanations can be suggested. Patients undergoing isolated ACLR included in our study might have had a lesser degree of anterolateral instability or anterolateral stabilizing structure injury. In contrast, those undergoing lateral augmentation in the form of ALL or LET might have had some degree of hyperlaxity, increased pivot shift degree, or performed in younger patients or as revision procedures, which are the main indications for lateral augmentation procedures in our institution. Furthermore, ACLR and ALL patients were not available for six and nine month follow-up, which may play a role in underestimating their effect on stability and clinical outcomes.

Concomitant meniscal procedures in ACLR have also been the subject of previous studies. In a retrospective analysis of 5378 patients by Cristiani et al., patients undergoing ACLR with or without medial and/or lateral meniscus resection or repair showed significant differences in the KOOS subscales at one and two year follow-up [13]. Likewise, in a prospective cohort study by the Scandinavian knee ligament registry, analysis of 8408 patients revealed no deleterious effects of meniscal injury or surgery on KOOS outcomes but significant improvement in KOOS pain, activities of daily living, and sport and recreation domains when compared to patients without meniscal injuries at five year follow-up [14]. Interestingly, significant differences in the preoperative scores were found among all groups, being those of the ACLR with medial and lateral meniscus repair with the lowest scores.

In contrast, when assessing IKDC scores after an ACLR with or without meniscus procedure, Cox et al. in a prospective cohort study found that medial meniscus repair or partial resection predict lower scores when compared to the absence of meniscus injury, but conversely, in the presence of lateral meniscus neglected tears and partial excisions, the scores were higher [15].

In our cohort, meniscal injuries were found in 57% of the patients, contrasting with previous publications with incidences ranging from 35 to 43% [16]. A more remarkable improvement in pain on activities was observed in those patients with ACLR and concomitant meniscal procedures at six week follow-up. Ulstein et al. showed that a higher preoperative pain on activities in the concomitant meniscus procedures group could explain this improvement [14].

Regarding lateral augmentation procedure complications, flexion contracture has been reported in 4.6% of patients undergoing ACLR with ALL [17], anterior knee pain in 7% [17], and hardware-related complications in 3 to 13% of patients as the most frequent complications [18, 19]. This is also supported by Na et al.’s meta-analysis, in which ACLR with LET increased the risk of knee stiffness and adverse events [11]. Although comparison of complication profiles was out of the scope of our study, we found a decrease of 22° flexion in the first six week follow-up in the ACL with ALL group. However, this difference was not observed in subsequent follow-ups.

The strengths of our study correspond to the comparison of two different lateral augmentation techniques of ACLR versus the isolated reconstruction in a predominantly athletic population. Our findings contribute to the current understanding of the role of lateral augmentation, especially during the early postoperative follow-up, which might be relevant in the quest for early return to sports. The present study certainly has some limitations. First, sample size calculations were not met, and the number of included patients corresponded to a lower inpatient flow due to the COVID-19 pandemic. Second is the follow-up’s short-term nature and the fact that the follow-up duration differs between the three groups. Moreover, some of the outcomes are subjective in nature, such as the subjective knee pain. In addition, there are differences between the three groups in terms of rate and type of concomitant meniscal procedures, in addition pivot shift grade. However, the findings of this study are of extraordinary interest due to the increasing popularity of lateral augmentation techniques and especially adverse outcomes derived from them. Moreover, procedures were performed by different surgeons, which might have had influenced the outcomes.

## Conclusion

ACLR with/without lateral augmentation procedures yields similar subjective IKDC, ACL-RSI, pivot shift grade, and subjective knee instability at short-term follow-up. The significant difference that was found at six week follow-up was the decreased flexion in the ACL with ALL group when contrasted to the isolated ACLR group.

## Data Availability

Not applicable.
